# Sexual Niche Segregation and Gender-Specific Individual Specialisation in a Highly Dimorphic Marine Mammal

**DOI:** 10.1371/journal.pone.0133018

**Published:** 2015-08-05

**Authors:** Laëtitia Kernaléguen, Yves Cherel, Travis C. Knox, Alastair M. M. Baylis, John P. Y. Arnould

**Affiliations:** 1 School of Life and Environmental Sciences, Deakin University, Burwood, Victoria, Australia; 2 Centre d'Etudes Biologiques de Chizé, UMR 7372 du CNRS-Université de La Rochelle, Villiers-en-Bois, France; 3 South Atlantic Environmental Research Institute, Stanley, FIQQ1ZZ, Falkland Islands; Sonoma State University, UNITED STATES

## Abstract

While sexual segregation is expected in highly dimorphic species, the local environment is a major factor driving the degree of resource partitioning within a population. Sexual and individual niche segregation was investigated in the Australian fur seal (*Arctocephalus pusillus doriferus*), which is a benthic foraging species restricted to the shallow continental shelf region of south-eastern Australia. Tracking data and the isotopic values of plasma, red blood cells and whiskers were combined to document spatial and dietary niche segregation throughout the year. Tracking data indicated that, in winter, males and females overlapped in their foraging habitat. All individuals stayed within central Bass Strait, relatively close (< 220 km) to the breeding colony. Accordingly, both genders exhibited similar plasma and red cell δ^13^C values. However, males exhibited greater δ^13^C intra-individual variation along the length of their whisker than females. This suggests that males exploited a greater diversity of foraging habitats throughout the year than their female counterparts, which are restricted in their foraging grounds by the need to regularly return to the breeding colony to suckle their pup. The degree of dietary sexual segregation was also surprisingly low, both sexes exhibiting a great overlap in their δ^15^N values. Yet, males displayed higher δ^15^N values than females, suggesting they fed upon a higher proportion of higher trophic level prey. Given that males and females exploit different resources (mainly foraging habitats), the degree of individual specialisation might differ between the sexes. Higher degrees of individual specialisation would be expected in males which exploit a greater range of resources. However, comparable levels of inter-individual variation in δ^15^N whisker values were found in the sampled males and females, and, surprisingly, all males exhibited similar seasonal and inter-annual variation in their δ^13^C whisker values, suggesting they all followed the same general dispersion pattern throughout the year.

## Introduction

The extent to which different individuals of a same population vary in their resource use is an important feature in foraging ecology. Resource partitioning at the population level has been observed in a wide range of taxa, particularly in the context of sexual segregation [1, 2]. Given that males and females often vary in their morphology, physiology and/or life history constraints, they might not have the same dietary requirements, activity budget and/or meet the same selection criteria in their respective foraging strategy. For example, sexual segregation in trophic niche is often associated with sexual dimorphism [3, 4]. Larger individuals (often males) have higher energetic needs, can potentially have access to a larger range of prey, or exclude conspecifics from favourable patches through dominance interactions [5–7].

Conspecifics of the same sex might also differ in their foraging behaviour [8, 9]. While individual specialisation is, in most cases, investigated at the population level, the degree of specialisation may vary within males and females [10, 11]. Indeed, when foraging in different habitats, upon different prey, or during different time periods, the two sexes may have access to a contrasting range of resources and experience distinct levels of competition and predation that will influence the degree of specialisation among individuals [8, 12, 13]. For example, in sea otters (*Enhydra lutris nereis*) females are restricted in their foraging grounds by the necessity to come back regularly to the breeding location to feed their offspring [11]. In areas of high population density, they experience a higher degree of intra-specific competition than males and exhibit higher individual variation in diet. However, only a limited number of studies has investigated gender-specific degree of individual specialisation within a population [10, 11].

Otariids (fur seals and sea lions) show extreme sexual dimorphism with males weighing two to four times the mass of females [14]. Furthermore, their reproductive strategy induces very different constraints on male and female foraging behaviour. Specifically, while males do not provide any parental care and can disperse after the mating period, females suckle their young for the duration of the lactation (4 to 36 mo) [15]. Hence, adult female otariids need to regularly return to their breeding colony to provision dependent offspring and are spatially constrained in foraging trip distance and duration by the fasting ability of their young. Accordingly, differences in foraging behaviour have been observed between the sexes in several species of otariids, both in terms of (i) habitat use (horizontal and/or vertical segregation), with males typically foraging further away from the breeding colonies and diving deeper than females [16–20], and (ii) diet, with males feeding upon larger prey of higher trophic level compared to females [10, 21, 22, 23]. As male otariids have potentially access to a wider range of resources than females, they should exhibit a wider trophic niche associated with a higher level of individual variation [8, 13]. However, comparison of the degree of individual specialisation between sexes have previously only been documented in two sympatric fur seal species, of which the study revealed contrasting results [10].

The Australian fur seal (*Arctocephalus pusillus doriferus*) is the largest fur seal species, with males and females weighing on average 279 kg and 76 kg, respectively [24]. Given the contrasting energetic requirements and constraints of the two sexes, sexual segregation would be expected in this species [3]. However, the local environment is also a major factor driving the degree of resource partitioning within a population [25]. In particular, resources need to be diverse enough to provide the ecological opportunity for conspecifics to diverge in their foraging niche. While the foraging behaviour of male Australian fur seals is poorly known, both males and females appear to be benthic foragers, restricted in their foraging habitat to the shallow continental shelf region of south-eastern Australia [26–28]. Therefore, this species represents an interesting model to investigate to what extent a highly dimorphic species, characterised with contrasting sexual breeding constraints, segregates at the sexual and individual levels, in a restricted environment. The degree of niche partitioning may vary in time, as constraints related to the annual cycle take effect (e.g. reproductive investment, migration) or the distribution and abundance of prey fluctuate [29, 30]. Hence, the aims the study were to investigate spatial and dietary sexual niche segregation in the Australian fur seal over different time-scales, and gender-specific individual specialisation over the long-term.

## Material and Methods

### Ethics statement

All procedures were conducted under Deakin University Animal Ethics Committee Approval (A14-2011, B12-2013) and Department of Sustainability and Environment (Victoria) Wildlife Research Permits (10005848). Fur seals were anaesthetised using tiletamine-zolazepam and/or isoflurane. Kanowna Island is part of the Wilsons Promontory Marine National Park and was accessed under permit from Parks Victoria.

### Animal handling and data collection

The study was conducted in June-July 2013, while males do not have any mating or breeding constraint and adult females suckle their single pup and, thus, need to regularly come back to the breeding colony (on average every 6.8 ± 0.6 d [31]). The Australian fur seal mating and pupping period occurs in November—December, and lactation lasts 10–11 months although some females may nurse a pup for a second or even third year [32]. Fieldwork was carried out on Kanowna Island (39°10’S, 146°18’E), northern Bass Strait, south-eastern Australia. Bass Strait is a broad area of continental shelf between Tasmania and the Australian mainland, characterised by a shallow and even bathymetry (average 86 m) [33]. The six largest and most accessible males present in the colony were chemically restrained using tiletamine-zolazepam (Zoletil, Virbac, France; 1.5 mg/kg estimated weight), that was remotely administered using 1.5 cc darts (Pneu dart) and a CO_2_ powered tranquiliser gun (Dan Inject JM Standard) [34]. Anaesthesia was maintained using isoflurane delivered via a portable gas vaporizer (Stinger, Advanced Anaesthesia Specialists, Gladesville, NSW, Australia). Six adult females suckling a pup were also captured using a modified hoop-net (Fuhrman Diversified, Flamingo, TX, USA), manually restrained and anaesthetised using isoflurane as described above.

Each seal was instrumented with a GPS data logger programmed to collect location data every 10 min (males: MK10AF, Wildlife Computers, Redmond, WA, USA; females: FastLoc, Sirtrack, Havelock North, NZ) in order to assess the degree of overlap in foraging areas between sexes, and a small VHF transmitter (Sirtrack) to assist with recapture. Instruments were glued in series to the dorsal mid-line fur just behind the scapula using quick-setting two part epoxy (Accumix 268, Huntsmen, Texas, USA), and devices were retrieved by cutting the hair beneath the glue at recapture. At the first capture, standard length and axillary girth were measured (± 0.5 cm). Females were weighed using a suspension scale (± 0.5 kg) while the mass of males was estimated from species- and sex-specific allometric relationship between body mass and morphometric measurements [35]. The longest whisker of each animal was cut as close to the skin as possible and a blood sample was collected by venipuncture of an inter-digital vein in a hind-flipper. Red blood cells were separated from the plasma fraction and all blood samples were stored at -20°C until isotopic analysis.

In the laboratory, whiskers were hand-washed in 100% ethanol and cleaned in an ultrasonic bath of distilled water for 5 minutes. Following Cherel et al. [36], they were dried, measured and cut into 3 mm-long consecutive sections starting from the proximal (facial) end. Plasma and red blood cell samples were freeze-dried and ground into a fine powder. Since lipids can affect plasma δ^13^C values they were removed using a cyclohexane solvent [37]. The δ^13^C and δ^15^N values of each whisker section and blood samples were determined by a continuous flow mass spectrometer (Thermo Scientific, Delta V Advantage) coupled to an elemental analyser (Thermo Scientific, Flash EA 1112). Results are presented in the conventional δ notation relative to Vienna PeeDee Belemnite marine fossil limestone and atmospheric N_2_ for δ^13^C and δ^15^N, respectively. Replicate measurements of internal laboratory standards indicated measurement errors < 0.10‰ for both δ^13^C and δ^15^N ratios.

### Comparison of the spatial and isotopic niche

The 95% and 50% Utilisation Distribution (UD) probabilities were calculated for the 35 day period when male and female foraging trips overlapped. The Utilisation Distribution Overlap Index (UDOI) [38] was then used to quantify the degree to which males and females shared foraging areas. Location data were filtered using a maximum swim speed of 8 m.s^-1^ to remove erroneous locations [39] and linearly interpolated every ten minutes. Smoothing parameters for the UD were calculated using the *ad hoc* method [40] and bathymetry data were used as an habitat grid to avoid unrealistic UD probabilities across land. The R packages trip and adehabitatHR [41] were used to perform these analyses.

Stable isotopes are increasingly used to studying the foraging ecology of wild animals [42, 43]. The δ^13^C values provide a proxy of a predator’s foraging habitat [42, 44], and δ^15^N values can be used to infer its trophic level [45]. Different time scales can be examined by using tissues with different turnover rates [46]. Although no turnover rates have been published for fur seals, plasma and red blood cells have half-lives of 4 and 28 days in black bear (*Ursus americanus*) [47], a carnivore of comparable size as Australian fur seal. In the present study, blood isotopic values were considered to be a proxy of the short- and medium-term foraging niche during the period just before seals were captured and tracked (winter). The otariid whisker is a continuously growing and metabolically inert tissue that retain its isotopic composition over time, such that serially sampled fur seal whiskers provide a chronology of δ^13^C and δ^15^N values over several years [10, 36, 48].

Short-term sexual segregation was examined by comparing male and female plasma and red blood cells isotopic values using t-tests after checking the normality of the data and the equality of variances. Long-term niche differentiation was investigated by examining the isotopic signature of seals’ whisker. Otariid whiskers show sexual and inter-individual variation in growth rate [10, 49] so that whiskers of similar length might not integrate a similar period of time. In order to compare groups and individuals over a similar time frame, specific whisker growth rates were calculated for each individual. Isotopic signature of otariid whiskers often present regular annual cycles along their length [10, 13, 36, 49, 50] which allows for growth rate to be estimated for each whisker. The periodicity of δ^13^C and δ^15^N values was assessed, using the wavelet analysis following Kernaléguen et al. [10]. This analysis allowed us to detect: (i) if the isotopic signature of whiskers consist of a repeated periodic signal; and, more importantly (ii) if the period of the cyclic pattern is consistent along the length of the whisker [51, 52]. As whiskers were cut and not plucked, their most recently synthesized tissue remained under the skin. This part of the whisker (not analysed) corresponds to an unknown period of time depending on its length and the specific growth rate of the whisker. As a consequence, the first section of each whisker does not necessarily correspond to the same time. Isotopic values were time-synchronised by doing a phase synchronization for each individual between its isotopic time series and the time series of a reference individual randomly chosen.

Variation in long-term isotopic niche between males and females was assessed by comparing the mean and range of whisker isotopic values between both sexes. Linear mixed-effect models were used to test the influence of sex (fixed effect) and individuals (random effect) on δ^13^C and δ^15^N mean values. The use of mixed-effect models allowed to account for the repeated measurements on the same individuals and the time correlation of whisker isotopic values (models included an auto-correlation factor) [53]. Differences in the estimated mean δ^13^C or δ^15^N values for males and females were tested using ANOVA analyses. Comparison of the range of δ^13^C or δ^15^N data between the sexes was then performed using *t*-tests after checking the normality of the values and the equality of variances. Comparisons were made over a period of 2.5 years which corresponds to the time period depicted by all whiskers.

Finally, the degree of individual specialisation in males and females was measured and compared using Roughgarden’s WIC/TNW index for continuous data [54]. Roughgarden [55] suggested that the population Total Niche Width (TNW, corresponding to the population variance) can be partitioned into the Within-Individual Component (WIC, intra-individual variance) and the Between-Individual Component (BIC, inter-individual variance), so that TNW = WIC + BIC. The WIC/TNW ratio is a measurement of the degree of individual specialisation: high values (approaching 1) indicate that individuals use the full range of the population resources, and low values (approaching 0) characterise specialist individuals. Each 3-mm long whisker section integrated an average period of time of 18 ± 5 and 38 ± 13 days for males and females, respectively (see Results) and was considered as one observation. Thus, WIC corresponded to the average variance between sections calculated at the whisker level and BIC to the variance between mean isotopic values of whiskers. The degree of individual specialisation was calculated on the most recent 2.5 years, a period of time depicted by all whiskers, so that all individuals contributed an equal weight to the analysis. The R package RInSp [56] was used to calculate individual specialisation indices. Significance of WIC/TNW was assessed using a nonparametric Monte Carlo technique to generate 10,000 replicate datasets under the null hypothesis that all individuals are generalists, from which P-values were calculated [54].

All results are presented as mean ± standard deviation and results were considered significant at the P < 0.05 level. All statistics were performed using R 3.0.3.

## Results

**Fig 1:**
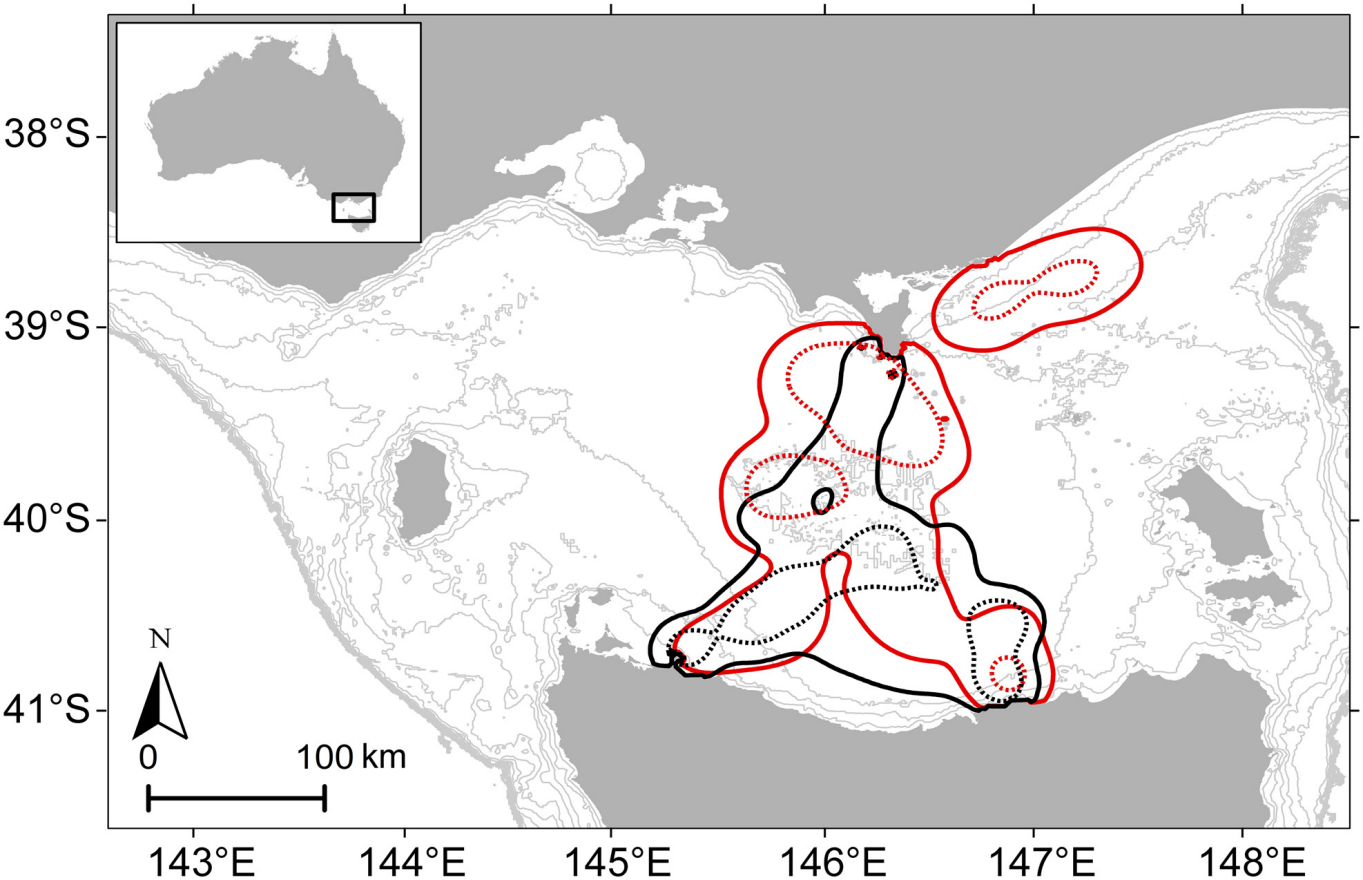
95% (plain line) and 50% (dotted line) utilisation distribution probabilities of males (black line) and females (red line), in June—early July (35 days). The black dot represents the breeding colony where seals have been captured and grey lines indicate the bathymetry (in 20 m intervals) to the edge of the continental shelf (200 m contour).

Males were significantly larger than females (Table 1) and had an estimated mass twice higher than females (138 ± 24 and 64 ± 6 kg, respectively, t_5.61_ = -7.41, P<0.001). Female foraging trips lasted on average 5.2 ± 6.5 d. Only half of the males returned to the breeding colony during the period they were tracked. The duration of the first trip performed by these three males was on average 22.5 ± 6.2 d. All individuals stayed within the continental shelf of central Bass Strait during the entire period they were tracked (35 days, from 4 June to 8 July) (Fig 1 and S1 File). Accordingly, both sexes displayed restricted home ranges that were within 220 km of the breeding colony, and exhibited spatial overlap in home ranges, with a 95% UDOI of 36% (Fig 1).

**Table 1:** Morphometric measurements and isotopic values of male and female Australian fur seals. Values are mean ± standard deviation.

	Males	Females	Statistics (P values)
Length (cm)	175 ± 13	145 ± 4	t_6.0_ = -5.34 (0.002)
Axillary girth (cm)	126 ± 8	100 ± 5	t_7.6_ = -6.89 (<0.001)
Mass (kg)	138 ± 24 *	64 ± 6	t_5.6_ = -7.41 (<0.001)
δ^13^C values (‰)			
Plasma	-19.4 ± 0.2	-19.4 ± 0.1	t_8.3_ = 0.36 (0.73)
Red cells	-19.0 ± 0.2	-18.9 ± 0.3	t_9.8_ = -0.69 (0.51)
Whisker	-16.8 ± 0.1	-17.1 ± 0.2	F_10,711_ = 9.32 (0.01)
δ^15^N values (‰)			
Plasma	16.1 ± 0.4	15.6 ± 0.3	t_9.8_ = -2.33 (0.04)
Red cells	15.9 ± 0.2	15.5 ± 0.3	t_9.4_ = —2.90 (0.02)
Whisker	16.8 ± 0.3	16.3 ± 0.4	F_10,711_ = 7.73 (0.02)

* Mass of males was estimated from species- and sex-specific allometric relationship between body mass and morphometric measurements [35].

**Fig 2:**
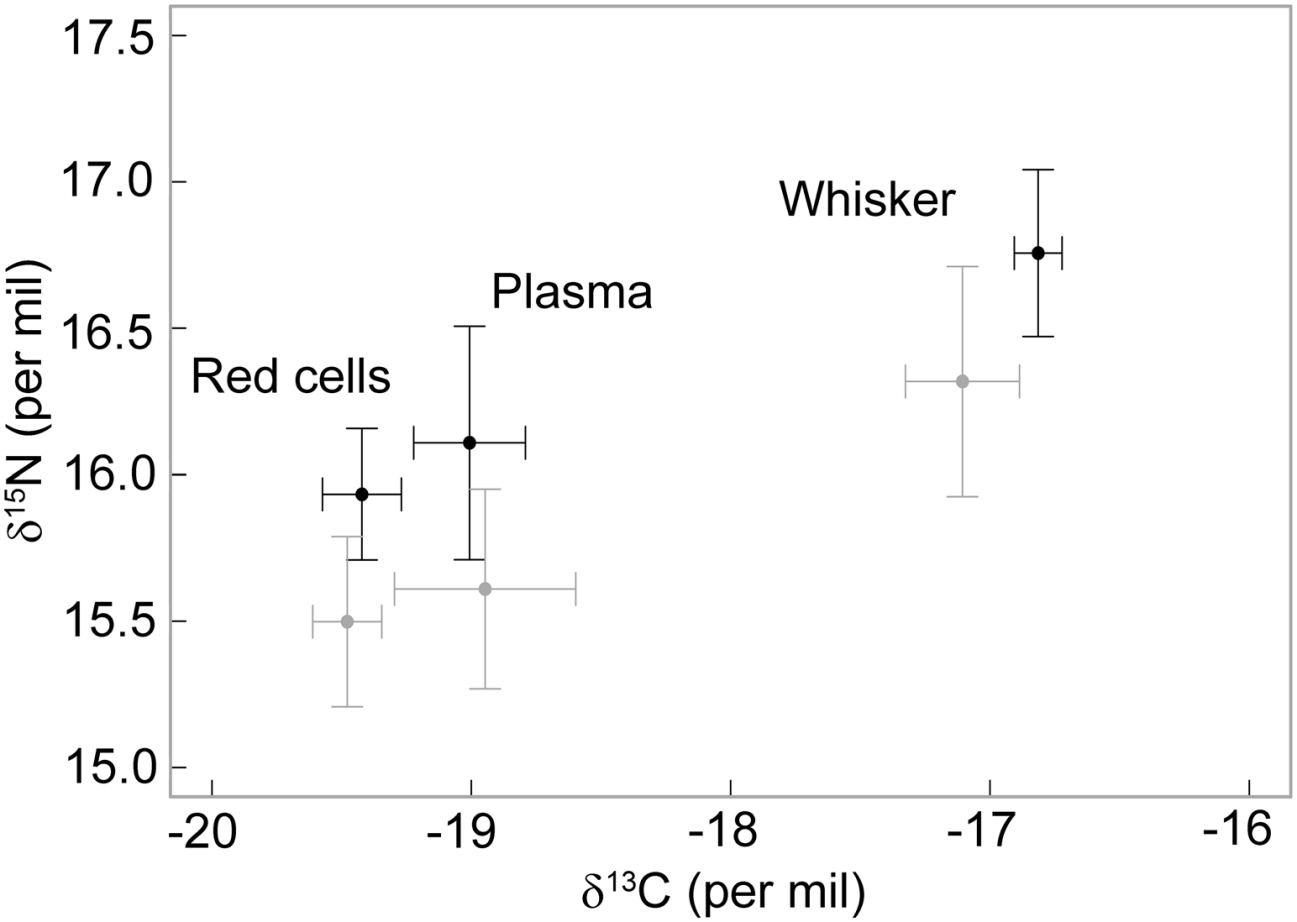
Plasma, red blood cells and mean whisker δ^13^C and δ^15^N values of male (black) and female (grey) Australian fur seals sampled in winter. Values are mean ± standard deviation.

Males and females exhibited similar average δ^13^C plasma (-19.4 ± 0.2 and -19.4 ± 0.1‰, respectively, t_8.3_ = 0.36, P = 0.73) and red blood cell values (-19.0 ± 0.2 and -18.9 ± 0.3‰, respectively, t_9.8_ = -0.69, P = 0.51) (Table 1, Fig 2 and S2 File). However, they consistently displayed a small but significant difference in their plasma and red blood cell δ^15^N values, with males having higher blood isotopic values than females (plasma: 16.1 ± 0.4 and 15.6 ± 0.3‰, respectively, t_9.8_ = -2.33, P = 0.04; red blood cells: 15.9 ± 0.2 and 15.5 ± 0.3‰, respectively, t_9.4_ = -2.90, P = 0.02) (Fig 2).

Male and female whiskers measured on average 210 ± 45 and 165 ± 30 mm, respectively. All seals exhibited constant periodic oscillations in δ^13^C and/or δ^15^N values along the length of their whisker. When both series (δ^13^C and δ^15^N) of a same individual were periodic (for five male whiskers), the period was similar for both δ^13^C and δ^15^N ratios. Whiskers recorded between 2.8 and 7.6 cycles, with an average of 3.5 ± 0.6 and 5.5 ± 1.5 cycles for males and females, respectively. Assuming cycles were annual [10, 13, 36, 50], average whisker growth rate was 0.17 ± 0.04 and 0.09 ± 0.03 mm·d^-1^ for males and females, respectively.

**Fig 3:**
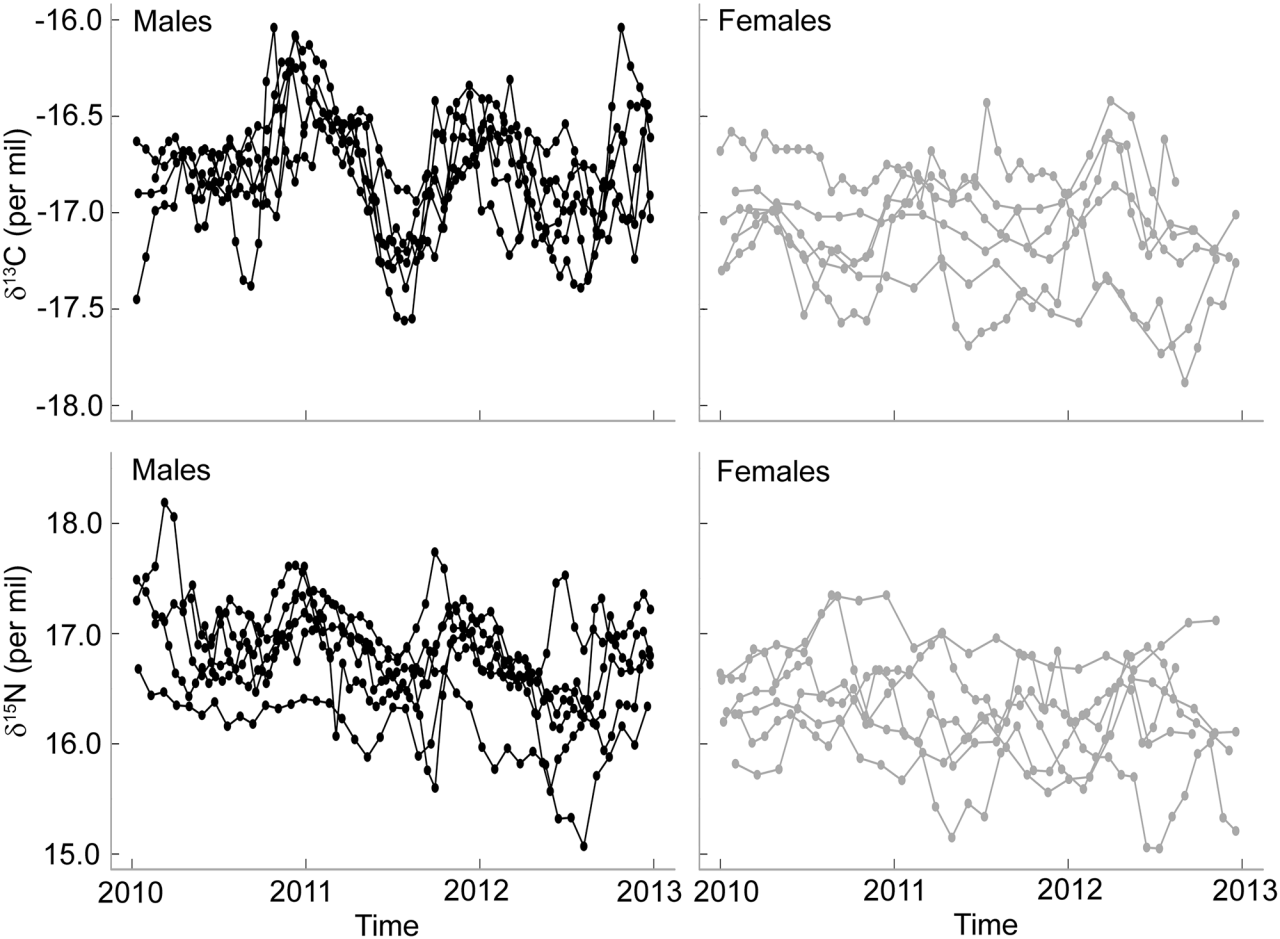
Whisker δ^13^C and δ^15^N values of male (black lines) and female (grey lines) Australian fur seals over three consecutive years.

A small but significant difference was found in mean δ^13^C and δ^15^N whisker values between males and females (δ^13^C: -16.8 ± 0.1 and -17.1 ± 0.2‰, respectively, ANOVA: F_10,711_ = 9.32, P = 0.01; δ^15^N: 16.8 ± 0.3 and 16.3 ± 0.4‰, respectively, ANOVA: F_10,711_ = 7.73, P = 0.02) (Fig 3). The range of whisker δ^13^C isotopic values was higher in males than in females (1.1 ± 0.3 and 0.7 ± 0.2‰, respectively, t_8.4_ = -3.59, P = 0.006), while it was similar for δ^15^N values (1.4 ± 0.2 and 1.3 ± 0.6‰, respectively, t_6.6_ = -0.38, P = 0.71, respectively). Both males and females exhibited significant individual specialisation. However, the degree of specialisation in males was very low (WIC/TNW = 0.93, P = 0.005) compared to females (WIC/TNW = 0.41, P<0.001) when considering δ^13^C values, and similar (WIC/TNW = 0.56 and 0.60, respectively, both P<0.001) when considering δ^15^N values (Fig 3).

## Discussion

The foraging niche of the sampled male and female Australian fur seals greatly overlapped, both in terms of foraging habitat (tracking data and δ^13^C values) and trophic level (δ^15^N values). As both sexes exhibit strong size dimorphism and face very different breeding-related constraints, pronounced sexual segregation was expected. However, if individuals’ traits (e.g. morphology, dietary requirements, breeding constraints) is a major factor driving resource partitioning within a population, the local environment may also play a fundamental role. In the present study, the uniformity of Bass Strait (shallow benthic foraging habitat) is likely to limit the opportunities for sexual niche segregation to occur.

### Spatial segregation

Tracking and δ^13^C isotopic data indicated a great overlap between male and female foraging habitats in winter. All study individuals foraged within central Bass Strait, a region characterised by a typically low primary productivity, particularly during winter [33]. As expected due to their central place foraging mode, females had a greater presence in the immediate area surrounding the breeding colony than males. As males do not provide any parental care, they were expected to disperse [27] to forage in more productive areas and avoid intra-specific competition with females. Surprisingly, even though males do not have any land-based dependent offspring, half of them returned to the breeding colony within the tracking period, potentially to gain knowledge of breeding territories and impose themselves into the hierarchy among males for future reproductive seasons [27].

The Australian fur seal is the most geographically restricted fur seal species, with a species distribution mostly limited to islands around the shallow Bass Strait region [57]. Numerous studies involving adult females have shown seals’ benthic foraging behaviour, limiting them to the continental shelf [26, 28, 58]. However, only a few studies have investigated the foraging ecology of males [26, 27, 59] and male diving behaviour has been recorded in only one individual [26]. Hence, it was not known if males could potentially display an epipelagic foraging behaviour and travel beyond the continental shelf. Even if restricted to the continental shelf, males could potentially have access to a wider range of foraging habitat than central Bass Strait. Indeed, Kirkwood et al. [27] found that adult males (n = 9) from a nearby colony (Seal Rocks) foraged mostly within Bass Strait but 33% of individuals also moved along the Tasmanian and/or South Australian coasts.

The isotopic values of whiskers integrate the foraging ecology of the same individuals over several years, documenting seasonal and inter-annual variation. As expected from the tracking and blood isotopic data, the δ^13^C isotopic values of male and female whiskers strongly overlapped, reflecting the similar winter foraging habitats. However, the study males exhibited greater intra-individual variation in their δ^13^C values than females suggesting they exploited a greater variety of foraging habitats throughout the year. In several otariid species, males are known to travel great distances just after the mating period, some species undertaking a yearly migration [10, 18, 36, 60]. Whereas in the present study male Australian fur seals remained close to the breeding colony in winter, the whisker δ^13^C values suggest they dispersed to different regions at other times of the year. In particular, they may move to more profitable areas just before and after the mating period, when they need to gain rapidly body mass in preparation to extreme fasting period (up to 60 days [24]), and to recover their body condition, respectively [27]. Indeed, even if the sampled males might not be reproductively active, unsuccessful bulls and bachelor males congregate in the vicinity of the breeding territories and spend long periods of time ashore fasting during the mating period [24].

While intra-individual variation observed in male δ^13^C whisker values is likely to reflect seasonal variation in foraging habitat, it could also denote potential seasonal variation of the isotopic signature of prey. Indeed, some prey are known to be migratory and enter into Bass Strait seasonally, potentially coming from different oceanographic regions characterised by different isotopic values. Furthermore, nutrient input of Bass Strait is primarily influenced by the Bonney Upwelling and three primary water masses, the South Australian Current Water, the East Australian Current Water and the sub-Antarctic Surface Water whose relative contributions vary across seasons and years [61, 62]. The baseline isotopic values of the trophic chains in these currents could differ such that the isotopic value of prey could vary over time. However, if males and females were foraging within the same habitat (i.e. within central Bass Strait) all year round, they should exhibit similar variation in their δ^13^C values. Hence, the potential temporal variation in the isotopic value of the food source cannot explain alone larger ranges of δ^13^C values in males.

### Dietary segregation

Males and females segregated in their δ^15^N niche over the long term (whisker isotopic data). Plasma and red blood cell δ^15^N values were also higher in males, indicating isotopic niche variation occurs even when both genders forage in the same habitat and have access to the same available prey (central Bass Strait, in winter). Therefore, difference in nitrogen isotopic values is not explained by potential spatial variation of prey distribution or isotopic signature. However, difference in δ^15^N values was small, with males and females overlapping in their isotopic niche. While this result suggests an overlap in the diet of the two sexes, it also reflects small variation in prey isotopic values. Indeed, in eastern Bass Strait, isotopic values of Australian fur seal prey species range between 11.5–13.9‰, with the main prey ranging between 11.5–12.8‰ [63]. Hence, even small variation in nitrogen isotopic values is likely to have a biological significance. The isotopic values observed for females in the present study are consistent with previous diet studies based on scat/regurgitate content analyses [64, 65] and animal-borne video cameras [66] indicating they feed mainly on benthic and bentho-pelagic fish (redbait (*Emmelichthys nitidus*), perch gurnards (Triglidae spp.), jack mackerel (*Trachurus declivus*), leatherjackets (Monocanthidae spp.), red cod (*Pseudophysis bachus*)) and octopus (*Octopus* spp.). The nitrogen isotopic values of males suggest they are likely to consume similar prey items than females, but a higher proportion of higher trophic level prey (e.g. larger octopus (*Octopus* spp.), barracouta (*Thyristes atun*), small sharks (Carcharhiniformes), stingrays (Myliobatiformes spp.)).

The degree of trophic segregation was surprisingly low compared to other otariid species [10, 21, 22] and what was expected in such a highly dimorphic species. Indeed, females are likely to be more limited in the prey size they can efficiently capture, handle and kill than their male counterparts. Conversely, their smaller body size may confer advantages over males with regard to manoeuvrability and the ability to capture small prey items [67]. Males and females also differ in their quantitative and qualitative energetic requirements. Males have higher absolute needs to maintain their larger body size. For example, in the Antarctic fur seal (*A*. *gazella*), males can have up to twice the energy requirement of females: 3.8 tons of krill a year for an adult male compared to 1.9 tons for a female [68]. Furthermore, as males are capital breeders and need large energy storage to prepare for long fasting periods during the mating season, they might be expected to primarily target prey of high lipid and protein content. In contrast, females are income breeders, forage throughout lactation and require different levels of lipid and protein for milk delivery [69]. Hence, both sexes should not necessarily share the same selection criteria in their respective foraging strategies and should accordingly show dietary differences. While the studied males were sexually mature and weighed approximately twice the mass of females, they were still not the size of prime territorial males. It is therefore possible that diet segregation will increase as body size continue to diverge with age [70].

### Gender-specific degree of individual specialisation

Female Australian fur seals are known to exhibit long-term individual specialisation in terms of foraging habitat and diet, with the population being comprised of both specialist and generalist individuals [66]. Given that males and females appear to differ in their diet and foraging habitat during certain times of the year (present study), both genders potentially experience contrasting levels of competition, resource availability or predation. As a result, the degree of individual specialisation might vary between the sexes [10, 11]. In particular, it has long been suggested that the accessibility of a greater diversity of resources (ecological opportunity, here foraging habitats) should favour individual specialisation, the variety of resources providing the raw material for inter-individual variation to occur [8, 71]. Accordingly, recent studies have found higher inter-individual variation when resource diversity increases, either temporally [13, 72] or spatially [25, 73, 74]. Hence, a higher degree of specialisation would be expected in males compared to females.

Surprisingly, the sampled males exhibited a similar degree of inter-individual variation in δ^15^N values and little difference in their δ^13^C values, when compared to females. When foraging in different habitats, males should encompass a spatial variation in prey assemblage and feed on a greater diversity of prey than females. However, male and female δ^15^N niche widths were similar and results suggest both sexes forage on a great variety of prey items, irrespectively of their foraging grounds. In accordance with the niche variation hypothesis which predicts that populations with wider niches exhibit more individual variability than populations with narrower niches [75], the high diversity of prey in seals’ diet was associated with a high degree of individual specialisation in both sexes.

The fact that males displayed very little inter-individual variation in their δ^13^C values suggests they all foraged in similar locations throughout the annual cycle. Considering males exploit a broader δ^13^C niche than females, a higher degree of specialisation was expected in males. This could indicate males go to specific productive habitats to feed. High density of prey would release intra-specific completion that should induce a decrease in the degree of individual specialisation [11, 13, 76]. However, this hypothesis assumes a high degree of competition in central Bass Strait. As for most cryptic species, it is difficult to quantify the degree of competition and its impact on the foraging behaviour in the Australian fur seal. The Australian fur seal is still slowly recovering from the severe over-exploitation of the commercial sealing era (1798–1825) and has not yet reached its carrying capacity [57]. Hence, this suggests food resource might not be a limiting factor, despite the relatively low productivity of Bass Strait [33].

In summary, while strong niche segregation would be expected in such a sexually dimorphic species, the trophic niche of the sampled male and female Australian fur seals showed a substantial degree of overlap. Yet, males exploited a greater diversity of foraging areas and fed upon a higher proportion of prey of higher trophic level than females. Seasonal variation in the degree of resource partitioning was observed, emphasizing the importance of studying resource partitioning over multiple time scales. The driving forces behind sexual dimorphism are still under debate [77]. The two main hypotheses developed are the *ecological divergence hypothesis*, where the morphology of males and females have diverged as a consequence of variation in the utilisation of prey resources by the two sexes [1], and the *sexual selection hypothesis* which predicts that dimorphism is a result of sexual selection that favour large, competitive males (or females) [78]. Results of the present study suggest the Australian fur seal is an interesting model to investigate the mechanisms of sexual segregation. Indeed, the sampled males and females showed a strong sexual size dimorphism, yet, overlapped in their respective trophic niches. The species distribution of the Australian fur seal is almost exclusively restricted to the Bass Strait region, implying all populations should face a similar restriction in their foraging habitats. If the observed overlap between male and female foraging niches is a generalized pattern across all populations, this would support the sexual selection hypothesis as the two sexes differ in their morphology but exploit similar prey resources.

## Supporting Information

S1 File Excel file containing the GPS location of all males and females during the tracking period. (XLSX)

S2 File Excel file containing the plasma, red blood cells and whisker isotopic values of each individuals. (XLSX)
